# *Taenia solium* neurocysticercosis: Its current epidemiological, diagnostic, therapeutic, and control landscapes

**DOI:** 10.1371/journal.pntd.0013937

**Published:** 2026-02-24

**Authors:** Javier A. Bustos, Christina M. Coyle, Kiran T. Thakur, Carolina Guzman, Luz M. Toribio, Gianfranco Arroyo, Herbert Saavedra, Kabemba E. Mwape, Vedantam Rajshekhar, Hector H. Garcia, for The Cysticercosis Working Group in Peru (CWGP)

**Affiliations:** 1 Center for Global Health, School of Health Sciences, Universidad Peruana de Ciencias Aplicadas (UPC), Lima, Peru; 2 Center for Global Health, Universidad Peruana Cayetano Heredia, (UPCH), Lima, Peru; 3 Cysticercosis Unit, Instituto Nacional de Ciencias Neurológicas, Lima, Peru; 4 Department of Infectious Diseases, Albert Einstein College of Medicine, Bronx, New York, United States of America; 5 Division of Critical Care and Hospitalist Neurology, Department of Neurology, Columbia University Medical Center, New York, New York, United States of America; 6 Department of International Health, Bloomberg School of Public Health, Johns Hopkins University, Baltimore, Maryland, United States of America; 7 Infection and Immunity Institute, St George’s University of London, London, United Kingdom; 8 Carrera de Medicina Veterinaria, Universidad Cientifica del Sur, Lima, Perú; 9 Department of Clinical Studies, School of Veterinary Medicine, The University of Zambia, Lusaka, Zambia; 10 Department of Neurological Sciences, Christian Medical College, Vellore, India; UDLA: Universidad de Las Americas, ECUADOR

## Abstract

Neurocysticercosis is the most common helminthic parasitic disease affecting the human central nervous system and is pleomorphic in its presentation. It is frequently encountered in daily practice in most parts of the world, and also commonly seen in industrialized countries in immigrant populations. In the past decade, new treatment (combined anti-parasitic drugs, increased attention to reducing treatment-associated inflammation and damage, new surgical strategies), and diagnostic (more specific antigen and antibody detection concepts and tools, more sensitive magnetic resonance imaging sequences) approaches, new animal models, and data on control of transmission have emerged. Still, diagnostic challenges persist and treatment approaches for some types of disease may differ, affecting clinical practice. This review provides clinicians in endemic and non-endemic countries with a comprehensive and practical reference to understand the variabilities in clinical expression of the disease and the optimal diagnostic and treatment approaches.

## Introduction

Neurocysticercosis (NCC), the infection of the human central nervous system (CNS) with the larval stage of the pork tapeworm *Taenia solium*, is considered a leading cause of acquired epilepsy worldwide and a significant contributor of neurological morbidity [1–3]. The life cycle of *T. solium* is maintained between humans and pigs in poor sanitation conditions and as such it is endemic in regions where pork is consumed. The clinical manifestations are protean, arising from a combination of factors that include location, burden, and stage of the parasitic lesions along with resultant host inflammatory response [4,5].

Cysts in the brain parenchyma are commonly associated with seizures. After cysts involute secondary to anti-parasitic therapy or natural involution, some resolve while others result in a calcified scar. Seizures can continue and emerge regardless of viability of cysts, including in calcified parenchymal disease. Extraparenchymal disease includes ventricular and subarachnoid NCC. Ventricular disease may cause complete, partial, or transient obstruction of cerebrospinal fluid (CSF) flow and associated ventriculitis. Subarachnoid NCC (commonly referred to as racemose cysticercosis), considered the most severe form of disease, causes hydrocephalus and intracranial hypertension, and carries the highest mortality rate if not managed properly [6,7]. In this narrative review we aim to increase awareness on the presence of the disease, and introduce readers to significant advances achieved in the past decade in the diagnosis (improved understanding of specific antigen and antibody responses and more sensitive magnetic resonance imaging [MRI] protocols), treatment (use of combined anti-parasitic drugs and a focus on reducing treatment-associated inflammation and damage as well as new surgical strategies), as well as novel animal models and confirmatory evidence of the efficacy of diverse control interventions to control the transmission of the parasite in endemic regions.

## Endemic regions

NCC represents a major public health challenge for many low- and middle-income countries where *Taenia solium* is endemic. Endemic areas include Central and South America, Caribbean, sub-Saharan Africa, and parts of Asia. In endemic regions, approximately a third of all seizures are caused by NCC [1,8,9]. The prevalence of NCC with epilepsy worldwide has been estimated at 4.36 million cases. The global burden of cysticercosis is estimated at 1.24 million (95% UI 0.78–1.81) DALYs. In Latin America and the Caribbean, cysticercosis holds the highest burden amongst neglected tropical diseases, with 295,000 DALYs (190,000–425,000) [10]. In Nigeria, disease burden in humans was 235,194.375 (95% uncertainty interval [UI]: 155,827.0–350,815.8) DALYs [11]. Recently reported community-based studies from Kenya [12], Uganda [13], Tanzania [14]. Côte d’Ivoire [15], Mozambique [16], and Burkina Faso [17] confirm endemicity in Sub-Saharan Africa. In China and Southeast Asia, NCC is still prevalent, but there is a marked scarcity of recent data [18–21].

Global estimates likely underestimate the burden of disease due to selection biases such as ignoring calcified disease NCC is still a major cause of seizures and epilepsy in endemic regions [22–25]; however, there are encouraging downward trends of NCC cases from some countries such as Mexico, Brazil, and Ecuador [26]. Anecdotal data suggests that the number of NCC cases seen in hospitals across India has also decreased significantly in the last decade (*V. Rajshekhar, personal communication, 2025*).

## Presence and impact in non-endemic countries

NCC is not restricted to settings close to the Equator or traditional tropical locations but is a global infectious disease of significant public health impact [1]. NCC is a disease of growing concern in immigrant populations in non-endemic regions (including in many high-income countries, where it was once deemed eradicated), where the disease often goes unrecognized. Australia, North America, Europe, and West Asian Gulf countries have seen a significant rise in NCC cases in the past few decades following increased migration from endemic countries and improved diagnosis [27–30]. Clinical cases in non-endemic regions mostly occur either among immigrants from or travelers returning from endemic regions [29,31–33]. More rarely, asymptomatic *T. solium* carriers entering new regions may cause autochthonous cases of cysticercosis without the need of infected pigs as evidenced by clusters of locally acquired cases in an Orthodox Jew population in New York reported in 1993 [34,35], and more recently in school kids in Belgium [36], demonstrating the potential for local transmission in non-endemic regions. NCC in international travelers traveling to endemic countries is thought to be infrequent, and most commonly associated with travel to these regions for prolonged periods of time. In one review of cases in international travelers, individuals became symptomatic at least a few years after returning home, and most commonly presented with seizures in the context of a single cysticercosis granuloma [37]. In addition, the worldwide pork market is growing exponentially, and is estimated to rise from approximately 250 million to over 450 million US dollars worldwide by 2028 [38]. Global pig production in rural communities has not been matched by efforts to enforce veterinary standards in pig husbandry. Expanded areas of pig husbandry with poor sanitation, free-range pigs, and lack of food safety governance significantly increase the risks of NCC expanding to novel regions.

## Lifecycle of the parasite

**Fig 1:**
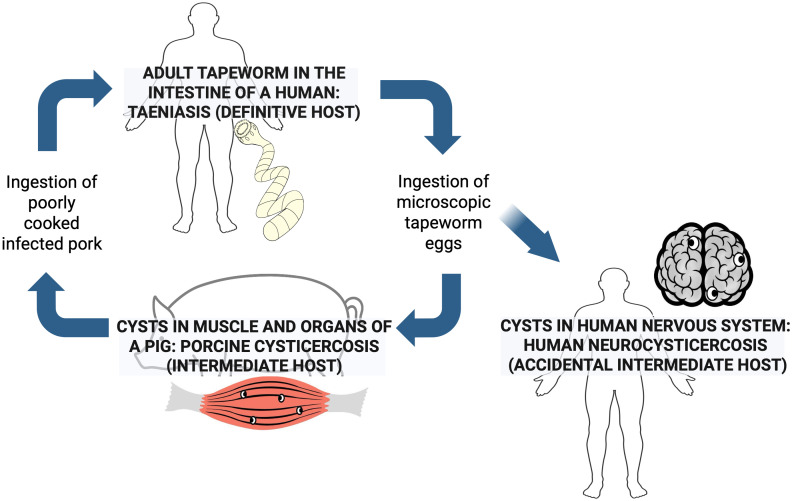
Life cycle of *Taenia solium.* Created in BioRender. Toribio, L. (2026) https://BioRender.com/39u5s15.

The lifecycle of *T. solium* is complex and involves two hosts, human, and pigs. Humans are definitive hosts and harbor the adult form (taeniasis), whereas pigs and humans can be intermediate hosts and harbor the larval form or cysticercus (cysticercosis) (Fig 1). Humans acquire intestinal taeniasis by ingestion of undercooked pork contaminated with cysticerci. The cysts evaginate in the small intestine, anchor to the mucosa, and develop into an adult tapeworm, which grows by forming new segments or proglottids from its neck region. As new proglottids are formed, the more distal segments mature and eventually are fertilized becoming gravid. Gravid proglottids containing infective eggs are detached from the distal part of the tapeworm and shed within the stool of a tapeworm carrier [39,40]. In areas with poor disposal of human stool and informal husbandry practices, pigs may access and ingest human stool containing *T. solium* eggs due to their coprophagic behavior [41]. The oncospheres contained in the eggs are released upon exposure to the acidic environment of the pig’s stomach and hatch in the small intestine after being exposed to the intestinal juices [39], penetrate the intestinal wall and enter the bloodstream, and are then distributed to the musculature and other organs (brain, eye, subcutaneous tissue) where they develop into cysticerci (larvae), which become fully mature in approximately three months [3]. Humans acquire cysticercosis mainly by accidental ingestion of *T. solium* eggs through the fecal-oral route if they are taenia carriers and do not wash their hands properly after cleaning themselves following defecation, or contamination of food and water with *T. solium* eggs, an event which is more likely in those who live in close contact with a tapeworm carrier.

## Natural history, pathogenesis, and clinical manifestations

Cysticerci in the subcutaneous tissue, muscles, or visceral organs either go unnoticed or cause minimal signs or symptoms. However, when cysts establish in the CNS, pathogenesis involves a dynamic interplay between the parasite and the host’s immune response. Viable cysts often suppress host immunity through mechanisms that downregulate T-cell activity via regulatory cytokines (IL-10 and TGF-β), impairing dendritic cells and macrophages [42]. Conversely, neuroinflammation drives the immune response upon cyst degeneration, in which released parasite antigens trigger a robust inflammatory response through activation of astrocyte proliferation and blood-brain barrier disruption to facilitate immune cell infiltration into the brain, leading to increased levels of pro-inflammatory cytokines. This immune activation leads to granuloma formation and edema, contributing to the development of clinical manifestations [43,44]. An excessive or prolonged inflammatory immune response can exacerbate neurological damage. Therefore, understanding these immunological dynamics is critical for developing therapies that balance parasite eradication with minimizing inflammation-related tissue damage.

The location of the parasite within the CNS is a crucial factor in determining the clinical manifestations of the infection [4]. While there are multiple syndromes of presentation of NCC, a simplistic but practical categorization separates infections in the brain parenchyma from those located outside the brain parenchyma (extraparenchymal NCC), namely ventricular and subarachnoid NCC (SANCC). Parenchymal cysts are primarily associated with seizures and epilepsy, although chronic headaches, cognitive, and psychiatric symptoms are also seen with some frequency [45]. Seizures are assumed to be of focal origin, and patients presenting with generalized seizures likely have focal onset seizures with rapid generalization rather than primary generalized epilepsies. The location and size of the cyst as well as the surrounding inflammation and damage contribute to the severity of the seizure disorder [46,47].

**Fig 2:**
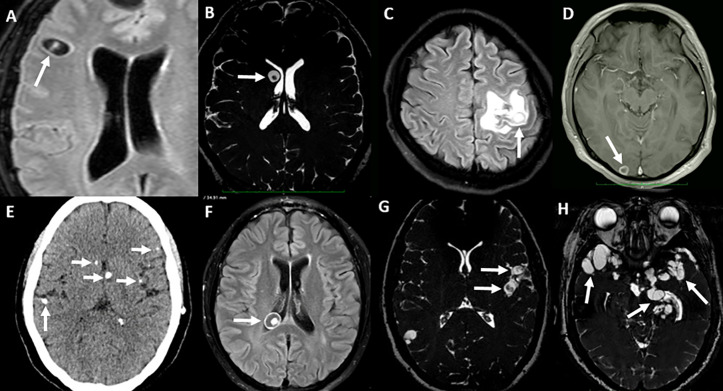
Parenchymal and extraparenchymal NCC. **(A–E)** Parenchymal neurocysticercosis—the process of degeneration of parenchymal cysts. **A.** Viable parenchymal cyst with no signs of inflammation. **B.** Early changes in the density of cyst contents. **C.** Marked pericystic inflammation with edema. **D.** Cyst collapsed to a ring-enhancing lesion. **E.** Calcified cysts on CT. **(F–H)**: Extraparenchymal NCC. **F.** Intraventricular cysticercosis. **G** and **H.** Subarachnoid NCC (G, well-defined cysts with visible scolices in the left Sylvian fissure [early subarachnoid NCC, arrows], H, Severe compromise of basal cisterns [arrows]). **A, C**, and **F** are FLAIR (fluid-attenuated inversion recovery) sequences; **B, G**, and **H** are balanced steady state sequences; **D** is a gadolinium-enhanced T1-weighted image, and **E** is a non-contrasted CT image.

Parenchymal cysts involute in a somewhat predictable manner which involves a perilesional inflammatory response. Initially, the cyst is surrounded by acute inflammation (that shows up in neuroimaging as contrast enhancement, following blood–brain barrier disruption) and edema, and then the cyst contents become turbid. The cyst subsequently collapses to form an inflammatory nodule which then resolves, but in some cases neuroimaging will reveal a residual calcification after a few months (Fig 2). Symptoms are more evident when the cyst begins to degenerate, but even after cyst destruction and calcification, symptoms such as seizures, headache, and cognitive impairment are frequently present [48,49].

Extraparenchymal NCC typically presents with mass effect, intracranial hypertension, vasculitis, and meningitis, resulting in hydrocephalus (headache, nausea, vomiting, papilledema), meningeal irritation, and stroke-like symptoms (motor and cranial nerve deficits, ataxia), with potentially fatal outcomes [50]. Interestingly, the membrane of the subarachnoid cysticercus can proliferate (contrary to what is expected for a parasitic cyst), which leads to an uncontrolled growth of the lesion with loss of the scolex, mass effect, and occupation of the surrounding spaces (Fig 2, images G and H). This process seems to involve a long latency period in the range of 10 and 20 years as shown by series of subarachnoid NCC in immigrants to non-endemic countries, or by the age difference in parenchymal versus subarachnoid cases in endemic regions [51,52]. The proliferating nature of the subarachnoid NCC membrane may provide enhanced repair capacity to the parasite in response to anti-parasitic treatment [53,54]. Host genetic factors may also influence disease severity or presentation [55]. A significant proportion of individuals with subarachnoid NCC also have parenchymal lesions, mostly calcified although viable cysts may also be seen.

There is a distinct difference between the clinical manifestations of NCC in patients from Asia and those from Latin America or Africa. Asian patients predominantly (nearly 80%) present with seizures whereas in Latin American or African patients a significant proportion of cases present with features of raised intracranial pressure (ICP) or meningeal form of the disease (SANCC) [56,57]. Also, cutaneous cysticercosis is more common in Asian patients than in Latin American patients [58]. The contributions of infection pressure, genetic diversity of the parasite or the host [59], cultural uses such as a vegetarian diet determining different ways of transmission [60], or different latency of some types of infection (SCG seems to have a very short latency period whereas subarachnoid NCC may present after more than 2 years of infection) [6] have not been defined. One of the most intriguing differences in clinical presentation pertains to parenchymal NCC which usually manifests in Latin American patients with multiple cysts at different stages of degeneration whereas a unique form of the disease with only a single degenerating cyst occurs in most Indian patients [56,61]. This form was recognized in the 1980s and 1990s as a single, small, enhancing CT lesion or a single enhancing lesion on neuroimaging studies [56,61]. Histopathological studies have identified these solitary lesions as solitary cysticercus granulomas (SCG) [62,63]. SCG is the predominant presentation of NCC in Indian patients with nearly two-thirds having this form of NCC [57]. Patients with SCG usually present with seizures, but some have episodic, severe headache resembling an ictal event, mimicking aneurysmal subarachnoid hemorrhage, as the only symptom [64]. The causes for these differences in disease presentation between patients from Asia and the Americas are not clearly understood.

## Diagnosis

The protean nature of the clinical expression of NCC makes it very difficult to arrive at a definitive diagnosis on the basis of clinical symptoms alone [65,66]. However, in endemic regions late-onset seizures (appearing after age 15–20) without other risk factors, or intracranial hypertension, are highly suggestive of NCC. Hematology and blood biochemistry analyses are usually not contributory. CSF is usually normal in parenchymal NCC, but high cellularity (predominantly mononuclear), high protein, and low glucose levels are typical findings in extraparenchymal NCC. CSF findings, in particular cell counts, are a direct marker of disease activity; however, CSF is not usually sampled in patients with extraparenchymal NCC due to increased intracranial pressure. Moreover, reluctance to perform lumbar puncture is quite common in poor settings where NCC is endemic [67–69]. Finding eosinophils in the CSF is quite suggestive of NCC, although not pathognomonic. The diagnosis of NCC is primarily based on neuroimaging and supported by immune and molecular diagnostic tests [4,70].

### Imaging diagnosis

Definitive diagnosis relies on the confirmation of the presence of parasitic lesions in the CNS by histology (rarely available) or neuroimaging techniques, such as magnetic resonance imaging (MRI) and computed tomography (CT). MRI provides more detail and can better define small parenchymal lesions and those close to the bone or the basal cisterns, as well as intraventricular cysts. It is also much more informative than CT with regard to parenchymal and meningeal inflammation (Table 1) [70,71]. However, MRI has poor sensitivity in detecting small calcified lesions [72].

**Table 1:** Role of the most common MRI sequences in the diagnosis and follow-up of NCC.

Sequence	Best suited for
**FLAIR** (*fluid-attenuated inversion recovery*)	Define parasitic lesions in particular content density. Define perilesional edema
**Balanced steady-state sequences** (FIESTA [*fast imaging employing steady-state acquisition*], CISS [*constructive interference in steady state*], BFFE [b*alanced Fast Field-echo*]	Define lesions in liquid-filled spaces (cisterns, ventricles, subarachnoid space). Define scolex in cystic lesions. Define liquid content of parenchymal lesions.
**Post-Gadolinium T1**	Demonstrate active blood–brain barrier breakdown/inflammation. Can be more useful than FLAIR to define small parenchymal lesions with inflammation
**Other sequences.** There may be a role for susceptibility-weighted sequences (star weighted angiography, SWAN; susceptibility weighted imaging, SWI, and venous blood oxygen level dependant, VenoBOLD) in detecting calcification although not as well as CT; Diffusion may help visualize the scolex; MR spectroscopy may help differentiating NCC from malignancy or tuberculoma, and magnetization transfer sequences can be useful to define residual gliotic areas left by resolved cysts.	

### Immunological assays

For many years, traditional immunodiagnostic techniques, principally using serum, have been applied as primary tools to support inconclusive imaging results and as a first screening test to identify NCC cases in low-income countries where neuroimaging is scarce outside major hospitals [8,73]. The meaning of an immunodiagnosis test result differs: antigen detection marks the presence of viable cysts, whereas antibody detection is useful for demonstrating past or present infection. Also, the performance of immunodiagnostic tests depends on the levels of the target molecule (usually much lower for antigen than for antibody), and these levels are in turn affected by the number and viability of NCC lesions [74]. As a consequence, a major drawback of these techniques is the low sensitivity (50%–60%) in patients with a single intraparenchymal cyst [75]. In addition, tests may be performed in blood, CSF, or other fluids such as urine, each with diverse advantages and drawbacks. Blood is commonly collected in most clinical settings and more tests are standardized for use in blood samples, CSF provides a direct access to the disease area, and urine is easily collected and non-invasive. On the other hand, systemic markers may not provide a direct assessment of CNS disease, and CSF collection is invasive and poorly accepted in most endemic regions.

The assay of choice for antibody detection, the Electroimmunotransfer blot using 7 lentil-lectin purified *T solium* glycoproteins (LLGP-EITB), provides sensitivity and specificity close to 100% for individuals with more than one viable brain cyst [76]. Antibody detection, however, cannot discriminate between active infections, past infections (as antibodies can persist for several years after cyst resolution), or even exposure to parasite antigens without established infection. Detecting circulating parasite-derived antigens confirms the presence of viable infections [74,77,78], and allows the monitoring of disease progression or responses to treatment [79,80]. Circulating antigen detection using ELISA (Ag-ELISA), based on genus-specific monoclonal antibodies (mAbs), has a sensitivity of 90% and specificity of 98.7% [74,77]. Several versions of Ag-ELISA involving different sets of mAbs exist, including B158/B60, HP10, and W5/W8 [81–83]. However, Ag-ELISA also fares poorly in patients with a single parenchymal cyst such as those with a SCG.

**Expected correlation between neuroimaging findings and serologic responses.** Wherever imaging and specific serology are available, the levels of antigen and the patterns of antibody bands should be compatible with neuroimaging findings and their results should be reasonably correlated. Some patterns of antibody response can be correlated with particular stages and types of NCC [74,84]. Positive circulating antigen should suggest the presence of viable cysts, and the levels of antigen and the patterns of antibody bands should correlate with neuroimaging findings.

CT and MRI both perform well in detecting multiple viable cysts, which are also associated with stronger antigen and antibody responses. In cases with only one or two viable cysts, CT findings may be subtle, whereas MRI provides better lesion visualization. In this setting, antigen may not be detectable, and antibody responses are generally weaker compared with cases involving multiple viable cysts. In individuals with only degenerating parenchymal cysts, antigen levels are typically very low or undetectable, while antibody responses may vary, also affected by the number of degenerating cysts. Calcified parenchymal NCC is more readily detected by CT than MRI and circulating antigen is usually absent, while antibody detection remains variable. Extraparenchymal NCC, both intraventricular and subarachnoid, is better visualized on MRI, whereas CT provides limited information. Subarachnoid NCC presents with very strong antigen and antibody responses due to greater parasite burden and increased host immune exposure.

Discrepancies between the serological test results and imaging findings should prompt more extensive patient assessment to identify alternative diagnosis or better define the intracranial disease.

Molecular tests were introduced nearly two decades ago [85] and more recently shown to perform well for the diagnosis and follow-up of extraparenchymal NCC [86,87], but their performance for parenchymal NCC remains to be defined. It is also unknown for how long detectable DNA can persist after the parasite dies [86].

### Limitations of existing diagnostic tools for NCC

Neuroimaging including CT and MRI machines are not universally available in developing countries and are especially scarce in poor rural regions where NCC is more prevalent. Similarly, the use of immunodiagnostic techniques to support NCC diagnosis is limited in regions with limited healthcare infrastructure and resources, due to the need for both parasite material to serve as antigens, and sophisticated equipment and trained personnel, making them high-cost techniques not affordable on a large scale.

### Advances in the last decade. New imaging protocols, new immunodiagnostic assays

In the past decade, the introduction of balanced steady-state MRI sequences such as FIESTA (Fast Imaging Employing Steady-state Acquisition) or similar ones has provided better definition of lesions in fluid-filled spaces such as the ventricles or basal cisterns [88].

The need for widely available, simple, reliable, and cost-effective techniques to facilitate the clinical decision-making and identify high-risk groups and communities has led to search for new immunodiagnostic approaches. Antigen-antibody dynamics have been deeply studied and characterized in different groups of patients and scenarios using LLGP-EITB and Ag-ELISA for antibody and antigen detection, respectively. Specific antibody response patterns and antigen levels have been associated with the type and stage of NCC [74,84].

The limited availability of native parasite glycoproteins limits their broader use. To address this, homologous recombinant proteins and synthetic peptides have been produced and tested in diverse diagnostic techniques [89–93], offering greater accessibility and reproducibility. As an individual diagnostic antigen, rT24H seems a promising alternative with sensitivities of 90%–100% in cases with multiple viable lesions [93,94], and also as a point-of-care test [95].

New approaches to investigate simultaneous antibody responses have been further developed with a multiantigen print immunoassay [96,97] and a Triplex-ELISA [98]. Antigen detection using Ag-ELISA based on B158/B60 mAbs has been further studied and validated [99]. Recently, new tools for improving antigen detection have been developed, such as anti-*T solium* specific mAbs targeting antigens from whole cysts (TsW8/TsW5 Ag-ELISA) [83,100], recombinant mAbs (TsG10) [80], and single-chain variable fragments (scFv) that recognize specific *T. solium* epitopes [101].

Similarly, a novel approach in using urine as a non-invasive sample to detect parasite products has been explored using Ag-ELISA [102] and a Point-of-Care (POC) test [103] offering rapid results for the identification of severe cases in rural settings. Molecular approaches have also been explored. Small circulating *T. solium* DNA fragments have been identified in urine (cfDNA), blood, and CSF using PCR and Loop-isothermal amplification (LAMP) [104]. The target sequences for these assays are mainly repetitive sequences from the *T. solium* genome such as pTsol9 [85,104], TsolR13 [87], and others [104]. Additionally, efforts in improving assay sensitivity for single NCC lesions included using a specific set of monocyte-derived genes [105]. Metagenomic next-generation sequencing approaches have been reported as well as a useful additional diagnostic tool in some difficult cases [106]. Although, several novel approaches to immunodiagnosis of NCC have been explored, none have become widely accepted or available. Even the EITB and Ag ELISA tests, which are considered the best available serological tests for NCC, are difficult to access or are very expensive and hence unaffordable in almost all regions endemic for NCC.

## Treatment

Though the disease was well described since the late 1800s, there was no specific agent to kill the parasitic cysts until the late 1970s. The only therapeutic options for cysticercosis were limited to surgery for cyst excision, ventricular shunts, antiseizure medications, or steroids to reduce inflammation. The discovery in Mexico that praziquantel (PZQ) was effective against porcine cysticercosis, and its subsequent use in human NCC, introduced the first effective cysticidal drug for this parasitic disease in 1978 [107]. PZQ and later albendazole (ABZ) [108] faced significant skepticism particularly from clinicians seeing populations with mild NCC presentations such as pediatric NCC and cases in the Indian subcontinent, where most affected individuals present with a SCG. It is now clear that the use of anti-parasitic agents to destroy parenchymal NCC cysts results in an improved clinical course in terms of fewer seizure relapses, and of SANCC after appropriate treatment is extremely rare [109–111]. In subarachnoid NCC, despite a few discrepant opinions, most experts recommend anti-parasitic treatment to reduce morbidity and mortality [4,8] (Table 2).

**Table 2:** Treatment options of choice by type of NCC. Types of NCC are ordered from the more severe to the less severe. In mixed cases, management should prioritize the most severe form.

Extraparenchymal	
Subarachnoid^*^	Anti-parasitic therapy until complete lesion resolution is obtained.^**^ Steroids should be initiated at high doses and gradually tapered with attention to relapses of inflammation or stroke
**Intraventricular**	Excision through minimally invasive surgery if possible
Parenchymal	
**Viable cysts**	Anti-parasitic treatment. Usually requires lower doses of steroids than subarachnoid disease
**Degenerating cysts**	Anti-parasitic treatment. Evidence seems to suggest a moderate benefit in lowering the resolution of seizure relapses and the chances of leaving a residual calcification
**Calcified cysts**	Symptomatic management only. There is no evidence that flares of peri-calcification edema would benefit from steroid treatment except in severe cases where it is causing intracranial hypertension or other symptoms due to the edema

* Cysts in the convexity of the cerebral hemispheres that are surrounded by brain parenchyma in most of their surface, behave as intraparenchymal cysts rather than as subarachnoid NCC.

** Monitored with neuroimaging and levels of parasitic antigen.

Interestingly, in the short term, anti-parasitic treatment of NCC produces acute inflammation as a result of the destruction of the parasite by the drug. This can lead to exacerbation or recurrence of existing symptoms such as raised intracranial pressure (ICP) or seizures or even the occurrence of symptoms which were not present prior to the therapy. Therefore, anti-parasitic treatment should not be expected to provide prompt relief from symptoms. It is essential to administer symptomatic (analgesic and antiseizure drugs) and anti-inflammatory therapies to the patient before and during anti-parasitic therapy. It is important to remember that NCC is a chronic disease and initiating anti-parasitic treatment is never a matter of urgency. Uncontrolled seizures and intracranial hypertension should always be addressed first and anti-parasitic agents should be delayed until the patient is stabilized.

While anti-parasitic drugs continue to be the cornerstone in the management of certain forms of NCC, treatment protocols have been refined over the past decade. To achieve optimal outcomes, the regimen to be used in an individual patient should be tailored to the location, stage, and number of cysts [8]. Anti-parasitic drugs (ABZ at a dose of 15 mg/kg/day in divided doses for 8–14 days with or without PZQ) are strongly recommended for patients with viable parenchymal NCC with imaging follow-up after three or six months of treatment onset. This recommendation is based on multiple studies including two placebo-controlled trials demonstrating more rapid radiologic resolution and fewer recurrent generalized seizures in patients treated with anti-parasitic drugs [109–111]. Other published trials showing no effect likely had major methodologic problems [112–114]. A SCG is the most common form of disease in India and US guidelines recommend treatment with antiparasitic therapy (weak recommendation) along with adjunctive corticosteroids (strong recommendation), also with imaging follow-up [4]. Given its natural involution, the benefit of anti-parasitic treatment in patients with SCG is less clear, although overall it seems to be associated with a lower risk for seizure relapses. There is no role for anti-parasitic agents when there are no viable parasites (e.g., calcified NCC). As mentioned above, steroids are used for their anti-inflammatory effect in conjunction with anti-parasitic drugs to counter the anticipated host inflammatory response to the death of the parasite. Prior to anti-parasitic treatment, all patients should undergo a fundoscopic examination to rule out ocular involvement.

The treatment of extraparenchymal disease should be approached in a very different manner than parenchymal disease. SANCC is due to a morphologically unique proliferative form of *Taenia solium* involving the subarachnoid spaces. There is a long incubation period suggesting that it takes years to develop with a resultant high parasite burden. Successful treatment requires prolonged or repeated courses of anti-parasitic therapy with concomitant steroids or alternative anti-inflammatory agents, with particular attention paid to avoid or control intracranial hypertension. CSF cestode antigen or cestode DNA falling to non-detectable levels predicts effective treatment along with resolution or stabilization of MRI [87]. Prolonged treatment with anti-parasitic drugs and steroids with extended follow-up has resulted in moderate disability and no mortality as reported in one series [6].

There are no randomized trials comparing different surgical strategies for the management of ventricular disease. These strategies include emergent control of acute high-pressure hydrocephalus by shunt placement, or other drainage procedures, followed by excision of cysts through craniotomy or endoscopic means. Neuroendoscopic excision of intraventricular cysts is frequently accompanied by endoscopic third ventriculostomy to address the associated hydrocephalus [115], but no controlled data exist in terms of efficacy, long term duration, or residual morbidity of this procedure compared to placement of a ventriculo-peritoneal shunt**.** Cysticidal treatment should be avoided prior to surgical excision of cysts as it can lead to cyst degeneration and adherence to the ependymal lining of the ventricles or to the surrounding parenchyma in subarachnoid NCC, making excision more difficult [116]. Corticosteroids are commonly employed to control the ventriculitis which may relieve intracranial pressure prior to intervention. Intra-operative rupture of the cysts during excision does not lead to a catastrophic anaphylactic reaction or spread of the disease and in fact, cyst rupture occurs almost always during surgery. No anti-parasitic therapy is required after complete excision of an intraventricular cyst unless there is concomitant disease outside the ventricles. As in the case of neuroimaging, availability of trained neurosurgeons, good surgical microscopes, and endoscopic devices is also lower in endemic countries.

### Combined ABZ + PZQ

ABZ is only partially effective against intraparenchymal NCC, and its performance is even lower in subarachnoid NCC [117]. Early publications reported an increase in efficacy when ABZ was combined with PZQ to treat cystic echinococcosis and parenchymal NCC [118,119]. More recently, two randomized trials by our group confirmed that this combination is significantly more effective in resolving multiple-cyst NCC [110,120]. In a study in Tanzania, combined ABZ and PZQ was 95% effective in eight patients who had previously failed ABZ monotherapy [121]. The concomitant use of PZQ significantly increases plasma levels of ABZ sulfoxide without increasing side effects [122,123]. The enhanced efficacy appears to result from additional synergistic effects beyond the increased plasma levels of ABZ sulfoxide. However, there was no benefit in combining PZQ with ABZ to treat NCC patients with only one or two lesions [110,120]. Recently, a study from India has shown better efficacy for combined ABZ and PZQ compared to ABZ alone in patients with SCG, leading a national expert committee to recommend its use in this type of NCC [124,125]. US guidelines recommend that PZQ should be added to ABZ in patients with more than two viable lesions as clinical trials have shown a significant benefit with dual therapy without increased side effects.

The combination of ABZ and PZQ is also commonly used to treat other types of NCC such as SANCC [6]. The efficacy of an initial course of anti-parasitic drugs in SANCC is significantly lower than in parenchymal disease. While the combination of ABZ and PZQ has been successfully reported in several case series of SANCC across various centers, data from controlled trials is still missing. Data from a controlled trial examining dual ABZ plus PZQ therapy in subarachnoid NCC will be available soon.

### Focus on inflammation

Most of the symptoms in NCC are due to the host inflammatory response and thus anti-inflammatory treatment is critically important in its management. For SCG or viable parenchymal NCC, typical steroid doses are prednisone 1 mg/kg/day or dexamethasone 0.1 mg/kg/day in divided doses, given during anti-parasitic therapy and slowly tapered after. A trial of higher doses of dexamethasone (8 mg/day for 4 weeks followed by a taper) was associated with fewer recurrent seizures during anti-parasitic treatment and early after dexamethasone cessation [126]. Interestingly, recent data suggests that the higher doses may also lead to fewer calcifications [127,128]. Whether the use of drugs to control inflammation can diminish the response to treatment has not been proven, but cannot be ruled out [129].

Prolonged anti-parasitic therapy in subarachnoid NCC induces host immune response and can lead to an inflammatory arachnoiditis which can result in hydrocephalus and vascular occlusion. [130–133]. Experts have used generous doses of corticosteroids with a slow taper over weeks to months during anti-parasitic treatment to avoid these complications, but long-term steroids are themselves associated with significant side effects [131]. More specific agents could provide improved options. Tumor necrosis factor (TNF) inhibitors such as etanercept have been used successfully as a steroid-sparing agent in one series, but further studies are needed [134]. SANCC is associated with a marked, CNS-localized cytokine-/chemokine-driven inflammatory response that largely decreases with curative therapy. The relative balance between proinflammatory and regulatory cytokines may be an important determinant for a cure in SANCC [6].

Despite advancements in antihelminthic therapy, ongoing seizures remain an issue for many patients with parenchymal NCC. Calcified NCC plays a large role in both the cause and maintenance of seizures and epilepsy in endemic populations [135]. The pathogenic mechanisms are probably multifactorial, but it is clear that inflammation in a subset of calcified lesions may be driving some of the neurologic symptoms observed in calcified granulomas. Symptomatic calcified lesions have been associated with perilesional edema and recurrent seizures in 35%–50% of cases. These lesions that serve as a seizure focus always reveal enhancement on T1-weighted post gadolinium MR studies. Experts have hypothesized that antigen is either sporadically released or recognized by the host, eliciting a local inflammatory response in the brain as evidenced by enhancement seen on MRI. In some patients, calcified lesions seem to be associated with hippocampal sclerosis and mesial temporal lobe epilepsy. This has raised the possibility of an inflammation-mediated mechanism to explain these observations, but clearly, further studies are needed [136].

### New minimally invasive surgical approaches

Surgery in NCC was for many years restricted to the placement of ventricular shunts or craniotomies to excise a single large cyst or cysts in the fourth ventricle. Endoscopy is now the option of choice for intraventricular NCC. There is wide experience around the world since 1998 [137], and multiple different surgical approaches have been used with success. However, surgical excision should proceed with caution when lesions show signs of inflammation (enhancement of the cyst wall on MR imaging and adhesion of the walls to the ependymal lining of the ventricle noted at surgery) since adherent lesions may bleed during the procedure. In such cases, a partial excision of the cyst wall is a reasonable option. In the case of SANCC, the existence of multiple separate parasitic lesions in different subarachnoid compartments of the brain, led to surgical excision not being considered as an option since multiple intracranial surgeries was not an appealing prospect. Recent advances in minimally invasive surgery techniques (neuroendoscopy, stereotaxy, microcraniotomies) have allowed relatively safe access and ability to excise large cysts from the subarachnoid spaces. This reduces the parasite burden which needs to be treated with anti-parasitic drugs [138,139]. Although no controlled data exist, surgically reducing the parasite load could have a beneficial effect in the form of reducing the intensity and extent of the post-treatment inflammatory processes.

A concise overview of diagnosis and treatment options in resource-limited countries compared to developed regions is provided below (Table 3).

**Table 3:** Diagnosis and treatment of NCC in resource-limited countries.

	Resource-limited settings	Well-resourced settings
**Clinical context**	Suspect NCC in late-onset seizures, intracranial hypertension, or atypical headaches. Initiate symptomatic management—do not wait for imaging or serology. Anti-parasitic therapy should not be initiated without neuroimaging..	Suspect NCC in late-onset seizures, intracranial hypertension, or atypical headaches in individuals coming from endemic regions or with a history of travel
**Imaging**	Limited CT availability, MRI rare, and limited by costs.	Whenever possible CT and MRI should be performed. MRI is more informative for active NCC, but CT is more sensitive for calcifications.
**Serology**	Specific assays (LLGP-EITB or derivate tests, monoclonal antibody-based ELISA for antigen detection) are rarely available. Low sensitivity and specificity of commercial antibody ELISA assays should be considered.	EITB should be requested to confirm etiology and antigen-detection assays (although scarcely available) to monitor persistence of viable infections.

## Animal models in NCC

**Fig 3:**
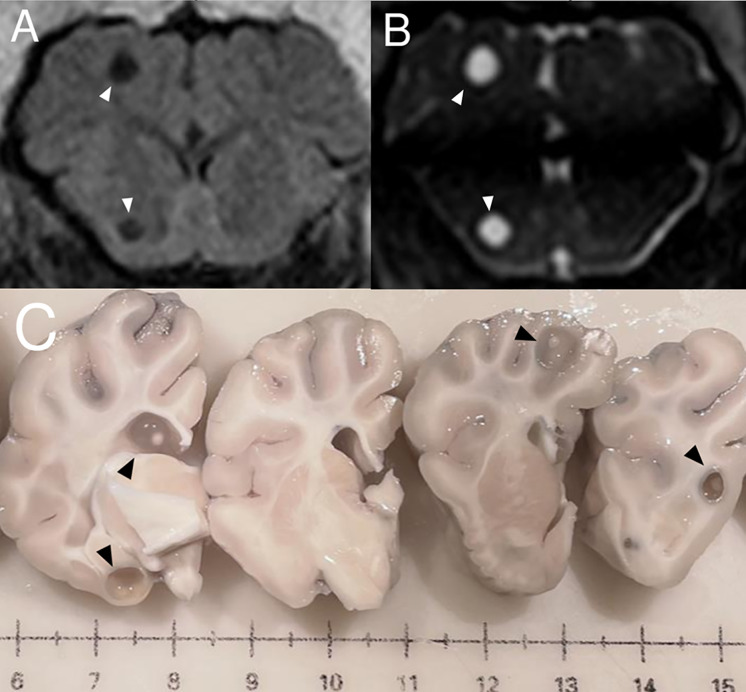
Neurocysticercosis in experimentally infected pigs. **A** and **B**. Coronal brain magnetic resonance images in FLAIR and BFEE sequences showing two well-defined intraparenchymal cysts. **C.** Macroscopic pathology showing viable brain cysts.

NCC models in rodents and pigs have been reported [140]. Rodents offer several advantages such as ease of handling large numbers of animals and the availability of laboratory reagents for rodents. Intracranial infection with *Mesocestoides corti* and *Taenia crassiceps* in mice have been used to characterize the granuloma formation and neuroinflammation [141,142]. Intracranial injection of *T. solium* oncospheres in rats successfully and consistently produce viable NCC and seizures with histopathological findings similar to those in humans [143,144], making it the most appealing rodent NCC model. Also, proliferative cestode larvae such as those of *Taenia crassiceps* may grow in the rat brain and simulate extraparenchymal NCC [145]. However, rodent models have drawbacks such as using infection routes that differ from the natural lifecycle, and a possible exacerbation of inflammation due to mass effects of the cysts in relation to their small brain size. Pigs are the ideal model for studying NCC since they are natural intermediate hosts of *T. solium* larvae and share similar brain anatomy and histological characteristics to humans. Naturally infected NCC pigs can be used [146,147], although uncertainties about cyst longevity, pre-existing inflammation, and the extremely high variability in parasite loads are important drawbacks in this model. Experimental oral porcine infection produces viable NCC but requires a high egg inoculum and lacks reproducibility [148–150]. Surgical implantation of activated oncospheres in pigs has been tested [151], but a significant percentage of CNS cysts are found in degenerating stages. A recently reported NCC pig model using intracarotid injection of *T. solium* oncospheres consistently reproduces viable NCC with a low brain cyst burden [152] (Fig 3) resembling human NCC and making it suitable for studying NCC and testing anti-parasitic and anti-inflammatory regimens in preclinical studies. Clinically apparent seizures are infrequent in pigs, although these have been shown to occur in pigs with severe NCC [153]. Potential uses of this model include to understand brain inflammation, damage, and epileptogenesis since, unlike the commonly used traumatic brain injury model, the extension and characteristics of the lesions are well-defined, and its evolution is highly predictable, and also the evolution of the lesions can be predictably modified by anti-parasitic drugs and/or anti-inflammatory and immunosuppressive agents. There is a possibility for other drugs to intervene to reduce calcification too. Other potential uses of animal models are to identify biomarkers for diagnosis and monitoring infection, cyst degeneration, or neuronal damage, and to improve anti-parasitic and anti-inflammatory treatment schemes [154,155].

## Control and potential elimination

As a neglected tropical disease, various strategies have been proposed over the years to control the transmission of *Taenia solium* [156]. These strategies encompass a range of approaches, including mass drug administration, sanitation initiatives, health education programs, rigorous inspection protocols, and pig vaccination, and need to be set in a One Health approach rather than individual initiatives. Improved sanitation is an evident need for *T. solium* control, but in most LMIC settings it is difficult to expect significant improvements in rural areas. Mass drug administration, targeted chemotherapy, and selective chemotherapy treatment programs directed at individuals have demonstrated evidence of efficacy and cost-effectiveness [41,157]. A large-scale intervention program in Peru involving human and porcine chemotherapy and porcine vaccination, provided proof of concept that *T. solium* transmission can be sustainably interrupted by active intervention, opening the possibility of eventual eradication [158]. In Sub-Saharan Africa, the best strategy for control and possibly elimination would be to include a combination of control options targeting the intermediate and human host [159]. However, the wide range of potential combinations and types of interventions (such as whole population versus focus-oriented) continue to pose challenges [160]. Mass drug administration of praziquantel faces the theoretical risk of triggering seizures in individuals with asymptomatic viable NCC as reported in multiple occasions. Although the risk seems low considering the large numbers of individuals receiving praziquantel for schistosomiasis in regions that are co-endemic for *Taenia solium*, it needs to be taken into account for program design and surveillance [157,161]. Compulsory notification of NCC cases, and even more of human taeniasis cases, can help policymakers to identify priority regions for intervention [162,163]. While these control measures are variably effective, the most obvious strategy to eliminate transmission of *Taenia solium* is universal implementation of sanitation measures and making communities open defecation-free. Unfortunately, good sanitation is still a distant dream in several endemic regions of the world.

## Conclusions and future directions

Neurocysticercosis remains a significant cause of neurological morbidity in vast areas of the world, and cases are now more frequently seen in industrialized and non-endemic countries because of travel and migration. Clinicians should understand the wide variability of clinical presentations of NCC and both the diagnostic and therapeutic approaches should be tailored to the specific type of NCC being treated. Unsolved needs in the diagnosis are the poor availability of neuroimaging in endemic regions and the poor availability of accurate and reliable immunodiagnostic tests worldwide. Sensitive tests are needed for detection of single NCC lesions, quantitative approaches for monitoring treatment efficacy, and cost-affordable and scalable tests to screen for severe NCC cases in endemic regions, including developing point-of-care tests for specific scenarios. Treatment should aim at complete resolution of viable cysts with the minimal possible inflammatory damage to the surrounding tissues. At a global level, a harmonized clinical data collection registry could provide base data for sound clinical and control initiatives. In the near future, data from CRISPR animal models for vaccine testing and artificial intelligence-based imaging tools can contribute to further advance NCC knowledge and prevention, diagnosis, and management.

### Search strategy and selection criteria

We searched PubMed, EMBASE, Web of Science, Global Health (CABI), and Global Index Medicus with search terms “cysticercosis,” “neurocysticercosis,” “*Taenia solium*,” or “taeniasis” for articles published from January 2000 onwards but before July 1, 2025, in English, Spanish, Portuguese, or Italian. Additional references were obtained from the personal archives of authors or manual searches of references from identified articles. The final reference list was generated on the basis of relevance to the main topic of this Review.

## Acknowledgments

Other members of The Cysticercosis Working Group in Peru include: Robert H. Gilman, MD, DTMH; Armando E. Gonzalez, DVM, PhD; and Victor C.W.Tsang, PhD (Coordination Board); Isidro Gonzalez, MD; Manuel Martinez, MD (Instituto Nacional de Ciencias Neurológicas, Lima, Perú); Manuela Verastegui, PhD; Mirko Zimic, PhD; Yesenia Castillo, MSc; Mónica J. Pajuelo, PhD (Universidad Peruana Cayetano Heredia, Lima, Perú); E. Javier Pretell, MD (Hospital Alberto Sabogal, ESSALUD, Callao, Perú); Eloy Gonzales-Gustavson, DVM, PhD; Maria T. Lopez, DVM, PhD; Cesar M. Gavidia, DVM, PhD (School of Veterinary Medicine, Universidad Nacional Mayor de San Marcos, Lima, Perú); Luz M. Moyano, MD; Ricardo Gamboa, MSc; Claudio Muro; Percy Vichez, MSc (Cysticercosis Elimination Program, Tumbes, Perú); Theodore E. Nash, MD; (NIAID, NIH, Bethesda, MD); John Noh, BS, Sukwan Handali, MD (CDC, Atlanta, GA); Jon Friedland (Imperial College, London, UK).
